# The Relationship between Empathy and Attachment in Children and Adolescents: Three-Level Meta-Analyses

**DOI:** 10.3390/ijerph19031391

**Published:** 2022-01-26

**Authors:** Xizheng Xu, Zhiqiang Liu, Shaoying Gong, Yunpeng Wu

**Affiliations:** 1 School of Psychology, Central China Normal University (CCNU), Wuhan 430079, China; xu_xizheng@126.com (X.X.); lzq200412@126.com (Z.L.); 2 Hunan Police Academy, Changsha 410138, China; 3 Center of Students’ Psychological Development, Yancheng Polytechnic College (YCPC), Yancheng 224000, China; 4 School of Teacher Education, Dezhou University, Dezhou 253023, China; wu_yunpeng@hotmail.com

**Keywords:** children, adolescents, empathy, attachment, meta-analyses

## Abstract

Empathy is one of the leading social abilities to understand or feel the emotions of other people. Attachment is thought to be a critical influential factor of empathy, as revealed by attachment theory and experimental studies, while empathy is also believed to facilitate the quality of attachment. Although many studies are conducted concerning the two subjects, the direction and magnitude of their relationship still remain unclear. In order to clarify the discrepant results in the previous study and explore the moderators in the empathy–attachment association, three-level meta-analyses were conducted in the present work. Based on 212 effect sizes from 59 samples in 50 studies with a total of 24,572 participants, random effect model analyses showed that empathy was insignificantly correlated with anxious attachment, significantly negatively correlated with avoidant attachment, and significantly positively correlated with secure attachment. The meta-analytic results indicated that children and adolescents with high secure attachment tend to show more empathy than those with low secure attachment. The meta-regression model revealed significant effects of the empathy dimension, culture, empathy measurement tools, and publication state. Additionally, implications and future directions for the empathy–attachment relationship were also discussed.

## 1. Introduction

Empathy is an important channel for health improvement and social adaptation of individuals, laying a foundation for moral development and positive outcomes [1,2]. A high level of empathy can promote prosocial behavior and the well-being of people while upgrading interpersonal relationships [3,4]. In contrast, a low level of empathy is closely related with the externalization of aggressive behavior and social adjustment [5]. Therefore, various internal and external factors affecting empathy have been extensively explored. Scholars often take attachment as an important antecedent variable [1,6]; other scholars also conducted intervention studies on fostering empathy [7]. In addition, empathy and attachment development in children and adolescents is a critical socialization content influenced by all kinds of individual and context variables [8]. Despite the fair amount of empirical data collected in the past studies, the empathy–attachment relationship and its direction and strength still remain unclear [9]. It is necessary to further clarify the relationship between empathy and attachment in children and adolescents through three-level meta-analyses based on the inclusion criteria of previous studies. The moderating variables that may affect the relationship between them should also be explored to determine the boundary conditions of their influence. The exploration of these problems will not only contribute to both the theories of attachment and empathy but also shed light on practices of empathy intervening measures.

### 1.1. The Concept and Measurement of Empathy

The concept of empathy has undergone a period of continuous construction with the emphasis on its single dimensions, such as affective empathy or cognitive empathy, to both dimensions [10]. Davis suggested that empathy is a kind of personality or a stable trait of feeling and understanding others’ emotions, including affective and cognitive factors. Affective empathy refers to emotional sharing in terms of other people’s situations, while cognitive empathy means emotional recognition and understanding [11]. However, some scholars believe that empathetic behavior, such as prosocial behavior owing to affective or cognitive empathy, is also a component of empathy [12]. Other researchers also point out that empathy can be divided into state empathy and trait empathy [6,13]. The former is caused by a certain situation, while the latter is a relatively stable individual difference. Regardless of the above diverse definitions of empathy, the two-dimensional component view of empathy, which consists of affective and cognitive sections, is widely accepted by most scholars [3].

Empathy measurement methods are diverse based on different definitions of empathy, including subjective and objective ones. Subjective measurements include questionnaire surveys and various behavioral evaluation methods. Then, the former can be divided into three categories. The first category is the affective empathy scale, including the emotional response scale (questionnaire measure of emotion empathy, QMEE) [14] and the empathy concern scale (ECS) [15]. The second category is the scale that measures cognitive empathy, including the Hogan empathy scale (HES) [16]. The third category is the scale that measures emotional and cognitive empathy, including interpersonal relation index (IRI) [17] and the basic empathy scale (BES) [18]. Objective measures of empathy include neuroimaging, electroencephalography, facial electromyographic activity, heart rate, and skin electrical response, which are mainly used on the study of state empathy [19]. In this study, empathy can be divided into affective and cognitive empathy from two dimensions. Empathy obtained from the scales that measure both emotional and cognitive empathy is called multidimensional empathy. 

### 1.2. The Concept and Measurement of Attachment

Attachment is the emotional connection formed between individuals and significant others in the early infant–caregiver interaction [20]. According to attachment characteristics, attachment is divided into different patterns, including secure attachment, anxious attachment (or contradictory attachment), avoidant attachment, and disordered attachment [21]. Early attachment studies mainly focus on the parent–child attachment. However, with the whole-life attachment theory and the multiple attachment theory arising, increasing attention has been paid to the attachment relationship between individuals and other significant figures, which is considered an essential part of attachment [22]. For the above reason, we classified attachment into parent–child attachment, peer attachment, and adult attachment, according to one’s past or current experiences with significant others, i.e., parents, brothers, sisters, friends, and partners [23].

In response to differences in attachment types and figures, various measurement methods have also been developed, including behavioral observation, story material, and questionnaire measures. Specifically, the behavioral observation method includes attachment Q-sort (AQS) and strange situation procedure (SSP) [24]. These two methods aim to measure children’s attachment sorts in real or experimental scenarios. Another type of measurement is performed by assessing children’s internal working models (IWMs) to determine their attachment styles, such as eliciting attachment-related dilemmas by telling stories and then asking children to solve those dilemmas [25]. The resolution of children to their dilemmas shows their IWMs, reflecting their attachment styles. The last category is the questionnaire survey. For example, the inventory of parent and peer attachment (IPPA) is wildly used for adolescence [26]. In addition, there are some other commonly used tools for measuring attachment, such as experience in close relationships (ECR) [27]. Although the attachment theory states that it has two aspects, the dynamic characteristics of the attachment behavior system and individual differences, namely state and trait attachment [28], the current study focuses on attachment as a stable trait.

### 1.3. The Relationship between Empathy and Attachment

The empathy–attachment relationship exists in three possible patterns. The first pattern is that attachment serves as the antecedent variables of empathy. The attachment theory reveals that infants develop their social emotions based on the attachment [19]. The infant–caregiver interaction is important for infants to recognize and understand the feelings and emotions of caregivers [20]. The theory also points out the specific mechanism by which children’s attachment quality affects empathy. Secure attachment enables children to develop positive IWMs, including self-affirmation and trust in others, which enable individuals to pay less attention to their feelings but more attention to others’ feelings [19]. Insecure IWMs involve the denial of the self and distrust of others, hindering individuals from understanding and feeling the emotions of others [29,30]. In addition, attachment also affects empathy through emotional regulation [31]. The second pattern is that empathy influences attachment. From childhood to adolescence, individuals develop peer attachment [32]. The equal status of individuals and their peers allows them to share and understand feelings for each other to meet their needs, thus maintaining and promoting peer relationships. This may further enhance peer secure attachment [33,34]. The third pattern is that there has been an interaction effect across time. According to the cascade model [35], empathy and attachment may influence each other in different development stages. The main pattern in early childhood is that secure attachment promotes empathy, while insecure attachment hinders empathy. In late childhood, especially in adolescence, empathy plays an important role in developing peer attachment. Based on the above theories and empirical research, we proposed:

**Hypothesis** **1.** *Empathy is positively correlated with secure attachment but negatively correlated with insecure attachment*.

### 1.4. Possible Moderating Variables

The inconsistent results of the strength and direction of empathy and attachment are caused due to various reasons. The heterogeneity of studies resulting from demographic characteristics, methods, instruments, and different theory bases are the main factors to induce the inconsistency. The analysis of these factors can explain the research gap to some degree. Thus, we take these factors into consideration in our meta-regression model.

#### 1.4.1. Gender

Previous studies indicated that empathy showed gender differences, namely, female empathy was greater than male [36], which was also reflected in the relationship between empathy and other variables. For example, studies found that the relationship between empathy and prosocial behavior was moderated by gender [37]. There were also gender variances in terms of the relationship between empathy and environmentalism [38]. Taken together, the above statement identifies gender may influence the strength of the empathy–attachment relationship. 

#### 1.4.2. Age

Although the attachment theory pointed out that the parent–child attachment formed in the early stage will continue to influence the individual’s psychology and behavior throughout life, there have been no clear conclusions as to whether the strength of this influence will change with age. In exploring the relationship between the attachment of children and adolescents to their mothers and self-control, researchers found that the former was significantly correlated with the latter, not in adolescence but in childhood [39]. The development rates of empathy and attachment are inconsistent in children and adolescents [40,41]. In early childhood, the single parental attachment may be transformed into multiple attachment relationships with multiple attachment figures formed in adolescence [42]. To sum up, the relationship between empathy and attachment in children and adolescents may present different patterns that continue into adolescence.

#### 1.4.3. Dimension of Empathy

The study proposed that the occurrence and development of cognitive empathy and affective empathy had different neural bases [43]. Cognitive empathy involves a series of cognitive processes influenced by children’s cognitive development, such as the theory of mind (ToM) and perspective taking. In contrast, affective empathy is more related to biological factors [44]. The above theories demonstrate that although cognitive empathy and affective empathy are related to secure attachment, they may have different influencing mechanisms. Affective empathy and cognitive empathy may have a unique influence on attachment, making multidimensional empathy the strongest contributor to the variances of attachment. Previous empirical studies also found that the cognitive and affective dimensions of empathy exerted different effects on various positive and negative behaviors. Generally, cognitive empathy had closer ties to other variables [45,46]. Therefore, cognitive empathy may be more closely related to attachment than affective empathy.

**Hypothesis** **2.** *Multidimensional empathy had the strongest correlation with secure attachment, followed by cognitive empathy and affective empathy*.

#### 1.4.4. Culture

Stern and Cassidy pointed out that culture is an important moderating variable affecting the empathy–attachment relationship [9]. Studies also found differences in empathy between eastern and western children and adolescents [47]. In addition, the difference in self-perception between eastern and western individuals also affects the effect of interpersonal relationships on empathy. In the context of the western individualistic culture, individuals regard themselves as an independent part, while against the background of eastern collectivism, the self is mainly defined according to one’s position in social relations and other people around them. This other-oriented self-perception makes easterners pay more attention to interpersonal harmony and others’ needs and closely connect their feelings with others to gain stronger empathy [48], which can further promote interpersonal relationships and enhance secure attachment. Based on the above views and empirical research, we put forward:

**Hypothesis** **3.** *Compared with those in western culture, empathy and attachment of children and adolescents are more closely related in eastern culture*.

#### 1.4.5. Attachment Figures

An individual’s attachment figures can be one or more, thus forming various attachment relationships according to the multiple attachment view. Views on the influence of attachment on different people during individual psychological development are varied. Bretherton pointed that the closest attachment partner (usually the mother) of a child is the most influential figure, and the attachment to whom is thus the best predictor of development outcomes [49]. However, some empirical studies found that peer attachment exerts greater impact on the adaptive ability of adolescents than parental attachment [50]. Other scholars have argued that all attachments are given equal weight and are thus integrated into a single IWM [51]. Opposite views are also available that multiple attachments are not integrated but form multiple independent IWMs with diverse functions in different developmental domains [52]. Given the inconsistency of views and the relative lack of research in the field, the question of whether attachment figures can moderate the empathy–attachment relationship will be explored.

#### 1.4.6. Other Covariates

Firstly, measurement tools originating from different comprehensions of empathy are applied merely based on the researchers’ understanding of empathy. Therefore, instruments for measuring empathy may lead to differences between empathy and attachment. Secondly, the concept of attachment is less controversial than empathy. Attachment measurement tools and methods encompassed in this meta-analysis are diversified. Differences in dimension division, number of items, and scoring method affect the research results. Thirdly, the questionnaire measures may lead to variation, including self-reported, parent-reported, and behavioral experiments. Finally, the publication status of the study and the reliability of the scale may also lead to variation.

### 1.5. The Present Study

As a further exploration of a recent review on the empathy–attachment correlation [53], the present study distinguished ToM and perspective taking that are different from empathy [54,55]. In addition, the previous study treated special groups, including patients and people who were attached to their pets, the same as normal children and adolescents, which may affect the accuracy of the conclusion. Those studies were excluded from the current study. The varied influence of different attachment figures on individual mind and behavior has always been controversial in attachment theory; this has not been analyzed. Therefore, analyzing the attachment figures as a possible moderating variable of empathy and attachment will clarify the boundary condition of the empathy–attachment relationship and enrich the attachment theory. In addition, different from previous analyses, the current meta-analysis adds important knowledge in several aspects. The specific measures in our study are as follows: (1) Chinese databases are searched to include more studies of Chinese subjects; (2) more studies on late adolescents are involved; (3) this study excludes non-empathetic elements from empirical studies, such as spatial perspective-taking and ToM on one’s values or motivation, and excludes non-human (e.g., pets) attachment studies to analyze the relationship between empathy and attachment more accurately; (4) the attachment figures factor is included as a possible moderator to examine the moderating effects of different attachment figures; (5) the study excludes patients with mental illness and other special subject groups to enable this study to be more applicable to ordinary children and adolescents. To sum up, the goals of this study include two aspects, specifically: clarifying the magnitude of the relationship of empathy and attachment, and finding the factors influencing the magnitude and direction of their relationship. 

## 2. Methods

### 2.1. Search and Inclusion Criteria

The full search process (including study selection and data extraction) documented and reported in this section conformed with the PRISMA guidelines [56]. We searched articles that were published up to the end of June 2021 through five electronic databases (Web of Science, PsycINFO, PubMed, Wanfant, and CNKI) with three categories of key phrases: (1) key words regarding empathy terms (empath*, cognitive empathy, affective empathy, social cognition, sympath*, social emotion, social skill*, ToM, and perspective taking); (2) key words concerning attachment terms (mother relation*, father relation*, parental relation*, peer relation* and attachment*); (3) key words relevant to children/adolescents (adolescen* or child* or infancy or youth* or toddler or teen*). In addition to electronic databases, we also traced reference lists of prior meta-analyses and systematic reviews on similar topics [53] and added potentially eligible studies for further coding. Referring to the age definition of adolescents in previous studies, the age of subjects in this study was limited to 21 years old [57].

**Figure 1:**
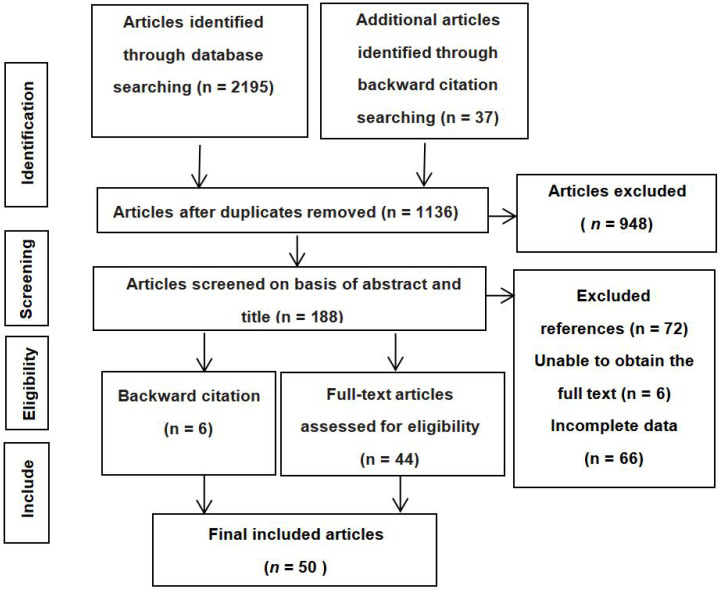
Flow chart of literature screening.

The following criteria were used to determine whether the studies were eligible for this meta-analysis: (1) The *r*-values of the relationship between empathy and attachment (the subdimensions of empathy–attachment relationship were also included) or *t*-values, *F*-values, or χ^2^ values were explicitly reported. The statistical magnitude obtained by regression analysis, structural equation model, and other statistical methods were excluded. (2) The sample size and the measurement tool were introduced. (3) Special population groups, such as children and adolescents with severe externalization problems and participants with various mental and non-mental disorders, were excluded. (4) Subjects aged above 21 were excluded. (5) Non-human attachment figures, such as animals, were sifted out. Finally, 50 articles (see Appendix A, Table A1) meeting the requirements were obtained, including 59 independent studies and 212 related effect sizes. The literature screening process is shown in Figure 1.

### 2.2. Coding the Studies

According to the widely recognized classification methods of the cognitive and affective dimensions of empathy and the trait empathy of children and adolescents concerned in this study, there were three kinds of empathy included in the literature: cognitive empathy, affective empathy, and multidimensional empathy. The specific coding methods were as follows. Firstly, two coders classified the empathy indicators involved in this study into cognitive empathy, affective empathy, and multidimensional empathy according to different measurement tools and item meaning. Secondly, the correlation coefficients of multidimensional empathy and attachment were coded separately. Finally, the study excluded ToM and perspective taking involving non-affective content, such as spatial perspective-taking [58]. Prosocial behaviors and willingness to help others were also excluded. Instead, only those studies relevant to the ToM and perspective taking that measured emotional understanding in the analysis were included.

Different attachment measurement tools lead to different dimensions of attachment (such as intimacy and alienation) [59]. All dimensions similar to the above were included in our study. Regarding the attachment figure, the attachment was classified into parental attachment, father attachment, mother attachment, and peer attachment in the meta-analyses.

Age was coded using the mean age of a sample, and gender was coded as the proportion of females in a sample. The studies’ country/region represents cultural differences that were classified and coded as western culture or eastern culture. Some countries that are difficult to classify were excluded from the coding. The measurement tools and published status were coded as dichotomous variables. The weighted mean of reliability of other studies was used as their reliability coefficient [60]. For studies using the experiment method, we set a reliability coefficient of “1” [61].

The first and second authors double coded the included articles with Kappa values (categorical variables) and internal consistency reliability ICC (continuous variables), ranging from 0.93 (attachment dimension) to 1.00 (sample size), indicating high coding consistency. For some studies, correlation analysis was performed on empathy and attachment, but only insignificant results were reported without concrete numbers whose correlation coefficients were coded as “0”. In this study, inconsistent coding contents were discussed to obtain consistent results. 

Finally, 50 studies were included in the current study, and 212 effect sizes from 59 samples were extracted. These studies were from 12 countries. The ages of the participants ranged from 2 years to 20.6 years. There were total of 24,572 participants, including 11,024 girls, and the mean age was 14.3 years.

### 2.3. Statistical Analysis

Because there was more than one effect size in some included studies, we conducted multilevel meta-analysis of skills to analyze the data, which can cope with the dependency of effect sizes [62]. Therefore, the method ensures that studies with more than one effect size and studies with one effect size have the same weight, except for sample size. There are three different variance components in three-level meta-analysis, as follows: (1) sampling error (level 1); (2) within-study variance (level 2); (3) between-study variance (level 3). Pearson’s correlation (*r*) was used as effect size in the current meta-analysis (first transformed to Fisher Z score for analyzing and finally back-transformed to *r* for the reporting of results).The rma.mv function in metafor package in the statistical software environment R 4.1.2 (R foundation for statistical computing, Vienna, Austria) was used to conduct the three-level meta-analysis [63,64]. The psychmeta package was used to correct the measurement error of effect sizes in original studies [65,66]. The corrected effect sizes were used in our analysis. Firstly, sensitivity analysis was conducted to detect the outlier of effect size. Secondly, three-level and two-level random effect models (including level 1 and 2 models and level 1 and 3 models) were established, and the different models were compared to determine the optimal one. Thirdly, we assessed the potential publication bias. Currently, there is no perfect method for detecting publication bias in a three-level meta-analysis. To address the effect size dependency, we randomly sampled one effect size per study and generated funnel plots. Asymmetry of funnel plot was tested by Egger’s regression test [67]. If Egger’s Z value was significant, the trim and fill method would be used to correct for the publication bias [68]. Fourthly, the pooled effect size was calculated. Finally, heterogeneity was assessed using Cochran’s *Q* and *I*^2^ statistic [68,69]. If the heterogeneity test index *Q* was significant or *I*^2^ was over 75% [70], then meta-regression analysis was used to test the moderating effects of various covariates, and the predictive power of the regression model was also calculated.

## 3. Results

### 3.1. Results of Sensitive Analysis and Model Comparison

Studentized deleted residuals (>2.5 are identified as outliers) and Cook’s distance were used to identify the outliers [71]. For the empathy and secure attachment model, the results showed two outlier effect sizes in our samples. When the outliers were deleted, the pooled effect size changed from 0.259 to 0.265, exerting little influence on pooled effect size. There were no outliers in the empathy and anxiety attachment model or the avoidance attachment model.

The attachment types included in the analysis consisted of three main attachment types: secure attachment, anxious attachment, and avoidance attachment. The model comparison indicated that the three-level random effect model fitted the relationship between empathy and secure attachment more significantly than the two-level model. The relationship between empathy and anxiety avoidance attachment. The three-level random effect model was significantly better than the model containing only levels one and three. Models that only had level 1, level 2 and level 1, level 3 had almost the same fit index. The comparison results are shown in Table 1. Lower index of AIC and BIC indicates a better model fit. Based on these model comparisons, we used a three-level random effect model to analyze the pooled effect size of empathy and secure attachment. The pooled effect sizes of empathy and anxiety attachment, as well as empathy and avoidance attachment, were analyzed using a two-level random effect model.

### 3.2. Results of Publication Bias Test and Pooled Effect Size

**Figure 2:**
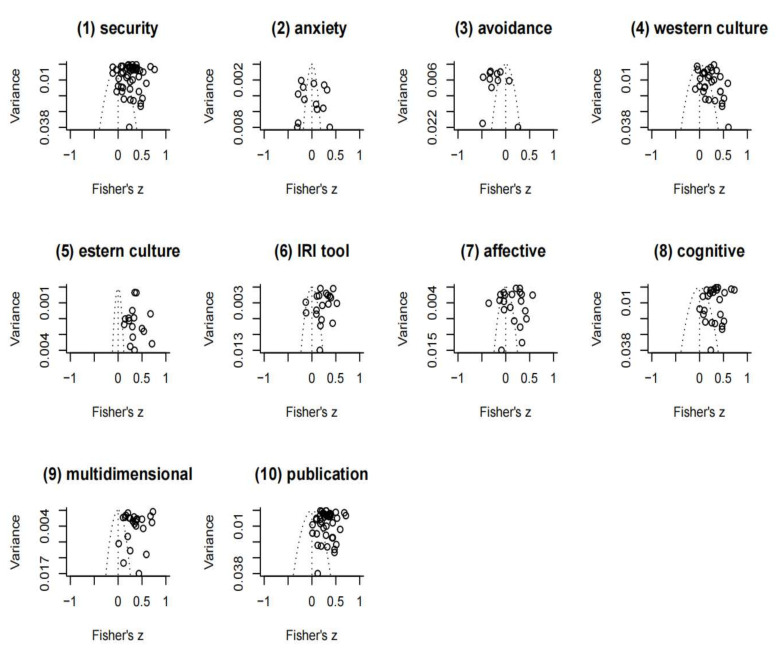
Funnel plot of Fisher Z scores. Note: (**1**–**3**) show models of dimensions of attachment type and empathy; (**4**–**10**) show models of empathy and attachment grouped by moderators’ categories.

From funnel plots (Figure 2), we can see the relative symmetry of the effect sizes of these 10 models. Further Egger’s regression tests were performed for each subgroup with more than 10 studies for a robust reason, respectively. The test results showed no serious publication bias in any group, indicating that publication bias had negligible effects on the meta-analysis results, as illustrated in Table 2.

From the analysis of the above model with 13 samples and 31 effect sizes, the correlation between empathy and anxious attachment was not significant, *r* = 0.034, 95% CI = (−0.101, 0.168). There was a significant correlation between empathy and avoidant attachment negatively, *r* = −0.103, 95% CI = (−0.195, −0.010), with 11 samples and 27 effect sizes. Due to the small number (<10) of covariates of avoiding attachment and empathy in the included study, no meta-regression was conducted. As a result, there was a positive correlation that existed between empathy and secure attachment, *r* = 0.259, 95% CI = (0.218, 0.299) (Table 2). These results partially supported hypothesis 1.

### 3.3. Moderating Analysis

For the three-level model of empathy and secure attachment, *Q*(*df* = 139) = 2751.56 (*p* < 0.001), *I*^2^ = 94.94%, indicated the reasonable random-effect model and the potential existence of moderating variables leading to heterogeneity.

In order to ensure sufficient statistical power of meta-regression, variables with fewer than 10 case numbers were excluded. Finally, only meta-regression analysis was performed on the empathy and secure attachment model. All the possible moderating variables were entered into the regression model simultaneously to detect their unique contributions (category variables were virtually coded, and continuous variables were centered). The results were as follows:

Empathy dimensions had a significant moderating effect (affective empathy as a baseline, *β*_cognitive empathy_ = 0.11, *p* < 0.01 *β*_multidimensional empathy_ = 0.11, *p* = 0.06). Specifically, the correlation between cognitive empathy and attachment was significantly higher than that between affective empathy and attachment. Other correlation differences were insignificant. Therefore, hypothesis 2 was partially verified.

Culture had a significant moderating effect (western culture as a baseline, *β* = 0.12, *p* < 0.01). Specifically, the empathy–attachment correlation in eastern culture was significantly higher than that in the western culture, which supported hypothesis 3.

Empathy measuring tools had a significant moderating effect. The correlation between empathy measured by IRI and secure attachment was significantly lower than that measured by the BES scale (BES as a baseline, *β* = −0.14, *p* < 0.05). Empathy measured by IRI and empathy task, empathy task, and BES led to no difference on the correlation between empathy and secure attachment (*p*s > 0.05).

The publication state also exerted a significant moderating effect (unpublished as a baseline, *β* = 0.13, *p* < 0.01), indicating that the published studies’ effect sizes were significantly larger than those in unpublished studies. The above results illustrated that the pooled effect size in this meta-analysis needed to be explained cautiously. Other covariates exerted no moderating effect (*p*s > 0.05). According to the above results, subgroup analysis was conducted according to empathy dimensions, culture, empathy measuring tools, and publication state, respectively (Table 2).

The *R*^2^ of the whole model was 0.30, meaning that 30% of variances could be explained by covariates referred to in this study; possible moderators influencing the relationship between empathy and secure attachment might still exist. 

## 4. Discussion

### 4.1. Empathy and Attachment

We conducted a comprehensive empirical review of the empathy–attachment relationship by performing a three-level meta-analysis and meta-regression. A total of 212 dependent effect sizes were analyzed, showing no significant correlation between empathy and anxious attachment. Ferguson et al. suggested that the generation of empathy requires cognitive cost [72]. Individuals maintaining that the cognitive cost of empathy is greater than the possible social reward (e.g., intimacy) will generate low or even no empathy motivation, according to the above view. Children and adolescents with anxious attachment show positive feelings toward others and negative feelings toward themselves. They still present great expectations for social rewards and high motivation to seek closeness and support for their attachment figures. However, the lack of confidence may prevent the expression of empathy and ultimately fails to establish relations between anxious attachment and empathy.

We found a low and significant negative correlation between empathy and avoidance attachment, consistent with previous studies [73,74]. According to attachment theory, IWMs with avoidant attachment showed self-affirmation and a negative attitude toward others. Avoidant attachment individuals manifested distrust of others and efforts to maintain their own behavioral and emotional independence [75]. Children and adolescents with an avoidant attachment would deny the social reward brought by empathy, be reluctant to empathize with others, and tend to avoid empathetic situations. This double lack of empathetic motivation and opportunity might hinder the development of empathy.

A significant positive correlation at a low to medium level was found between empathy and secure attachment, supporting the attachment theory on the influence of secure attachment on empathy [9]. Adolescents and children had high-quality parental attachment, and attachment to peer showed more empathy to their peers. Furthermore, although our study included different studies from previous meta-analyses, the above correlation magnitude was quite similar to the meta-analysis of Li et al. (*r* = 0.27), indicating the robust relationship magnitude of empathy and secure attachment. In addition, this correlation also indicated that multiple factors might influence the development and variance of empathy or attachment, which cannot be fully explained by a single factor.

### 4.2. The Influence of Covariates

No gender difference in empathy–attachment correlation was found in our study. Furthermore, recent meta-analyses of correlation between empathy and parent–child–peer relationship also failed to identify the moderating effects of gender [46]. The gender similarity hypothesis revealed that males and females did not differ in the extent of most psychological variables [76], which was supported by our study somewhat. There was also no significant difference in the relationship between empathy and secure attachment in children and adolescents at different ages, consistent with a recent meta-analysis [53]. 

The secure attachments of parents, fathers, and peers were equally correlated with the empathy of children and adolescents. However, previous studies on empathy and interpersonal relationships found that empathy was more closely related to peer relationships than parental relationships [46], which was different from this study. These conclusions suggested that empathy and secure attachment may have interaction mechanisms different from relationship and empathy.

The empathy dimension had a moderating effect on the relationship between cognitive empathy and secure attachment, which was significantly greater than that of affective empathy and secure attachment. The finding was consistent with previous studies on cognitive empathy and affective empathy [46,53]. Neurodevelopmental studies found that the development of affective empathy was consistent with that of the limbic amygdala, a brain region related to autonomic nerves. However, the development of cognitive empathy was consistent with that of the prefrontal cortex, a brain region related to higher cognitive functions [77]. The IWMs formed by attachment were processed and stored in the implicit memory system of the right cerebral cortex, highly consistent with the brain regions relevant to cognitive empathy activities [78]. This more similar physiological basis might also lead to a higher correlation between psychological functioning and cognitive empathy. These findings provided empirical support for further deepening the mechanism of attachment theory on how secure attachment affects empathy through IWMs. Although multidimensional empathy and secure attachment had the largest pooled effect size, they showed no significant difference between cognitive empathy and attachment. Therefore, these results should be enplaned cautiously.

The correlation between empathy and secure attachment in children and adolescents in eastern nations was significantly higher than those in western nations. Differences in socialization modes that exist between eastern and western children and adolescents may be one reason for this result [79]. For example, Lu found that eastern children can promote their ToM when talking about others, while western children realize this when talking about themselves [80]. In addition, such differences in socialization modes are also reflected in the relationship between attachment and peer relationships. For example, a meta-analysis showed that the correlation between attachment and peer relationship is weaker in north American countries that represent western culture than in other countries [59]. 

A significant moderating effect of measurement tools suggested that system errors might be induced by study methods. For the moderating effect of empathy measurement tools, the correlation between empathy measured by IRI and secure attachment was significantly lower than that measured by other scales. Studies based on IRI pointed out that in affective empathy, more empathetic concern represents more positive empathetic development. A high level of personal sadness means a negative development of affective empathy, weakening the relationship between affective empathy and secure attachment. In addition, there is usually a low correlation between the imagination dimension in the IRI scale and attachment, reducing the correlation between cognitive empathy, multidimensional empathy, and secure attachment.

Although the study failed to prove a moderating effect of gender and age, it found that the moderating effects of empathy dimension, culture, empathy measurement tools, and publication state enhanced the understanding of the boundary conditions of the relationship between empathy and secure attachment to some extent. Furthermore, the insignificant moderating effect of attachment figures verified the theory of attachment that the IWMs became relatively stable as children grew up to some degree. The current results also provide a useful reference for educational practitioners to implement targeted interventions for improving attachment and enhancing empathy.

## 5. Research Deficiencies and Prospects

Although the current meta-analyses have clarified the empathy–attachment relationship for children and adolescents, limitations are unavoidable. Firstly, the studies included in this work were not comprehensive enough (e.g., six studies couldn’t be obtained). Whether the results can be generalized to more people remains to be tested. Future studies should include more relevant studies to obtain in-depth and consistent conclusions. Secondly, the age distribution of subjects and the number of studies on variable measurement methods were relatively unbalanced. For example, the study subjects were mainly adolescents, and most studies adopted the self-report method. Therefore, conclusions were susceptible to the common method deviation, affecting research results. In the future, more objective measurement methods should be adopted to strengthen the accuracy of results from studies on empathy and secure attachment of children, especially preschoolers. Thirdly, moderators employed in our study were insufficient to referee the *R*^2^ of the regression model. Other possible moderators might have been neglected. Fourthly, since most of the literature included in the meta-analyses came from cross-sectional studies, the findings failed to reveal causality. In addition, attachment can influence empathy in various ways [9]. Children with high empathy are more likely to form secure attachments with parents and peers by following a kind of cascading model, developing a long-term interaction process [35]. Therefore, more longitudinal study design and experimental design should be combined with effective measurement methods to reveal the causal relationship between them. Finally, the outcome variables influenced by empathy and attachment, such as anxiety and suicide in adolescents during the COVID pandemic, should be exploded. Furthermore, whether and how public policy, such as COVID confinement, influences adolescents’ mental health should also be further studied [81,82].

## 6. Conclusions

The present three-level meta-analyses tested the relationship between empathy and attachment in children and adolescents, clarified the magnitude of the relationship between empathy and attachment, and also found some influential factors, such as empathy dimension, culture, measuring instruments, and so on. The results demonstrated that: (1) There was significant positive correlation at the low to medium level between empathy and secure attachment, and a low significant negative correlation between empathy and avoidant attachment. The relatively low or no effect size are not in line with some scholar’s expectation [9] of the role of attachment on empathy, which may lead theorists to change the way they explain empathy in terms of attachment theory. (2) The empathy–attachment relationship of children and adolescents was moderated by empathy dimensions. The correlation of cognitive empathy and secure attachment was higher than that of affective empathy. (3) The correlation between empathy and attachment in children and adolescents is weaker in western culture than in eastern culture. (4) Empathy measurement tools and the publication state can significantly influence the relationship between empathy and secure attachment.

## Figures and Tables

**Table 1:** Comparison between the three-level random effect model and the two-level random effect models of empathy and attachment.

Models	Empathy and Security Attachment			Empathy and Anxious Attachment			Empathy and Avoidant Attachment		
	AIC	BIC	LRT	AIC	BIC	LRT	AIC	BIC	LRT
Model 1	−47.83	−38.73		20.65	24.85		5.65	9.43	
Model 2	1111.83	1117.89	1161.65 ***	20.01	22.82	1.37	3.66	6.17	0.00
Model 3	−38.76	−32.70	11.06 ***	337.56	340.36	318 ***	100.69	103.20	97.03 ***

Note: *** = *p* < 0.001; Model 1 = three-level model; Model 2 = two-level model including level 1 and level 2; Model 3 = two-level model including level 1 and level 3; AIC = Akaike information criterion; BIC = Bayesian information criterion; LRT = likelihood ratio test.

**Table 2:** Analysis of correlation and moderating variables between empathy and secure attachment.

Models	Egger’s Z Value	k	n	Q	*r*	95% CI	Level 2 *I*^2^	Level 3 *I*^2^
1	0.356	51	154	2751.56 ***	0.259	[0.218, 0.299]	69.90%	25.05%
2	0.761	13	31	745.44 ***	0.034	[−0.101, 0.168]	78.11%	17.64%
3	0.067	11	27	267.67 ***	−0.103	[−0.195, −0.010]	90.33%	0.91%
4	1.214	16	58	1244.89 ***	0.313	[0.235, 0.387]	44.12%	52.99%
5	0.277	31	90	1435.29 ***	0.212	[0.161, 0.262]	80.82%	9.50%
6	−1.429	24	44	820.84 ***	0.328	[0.265, 0.388]	49.86%	43.38%
7	−0.733	25	51	327.12 ***	0.260	[0.196, 0.321]	57.36%	30.85%
8	−0.724	21	59	776.56 ***	0.163	[0.109, 0.216]	91.47%	3.53%
9	−1.40	19	85	1203.01 ***	0.185	[0.135, 0.234]	79.08%	15.67%
10		6	20	593.06 ***	0.367	[0.274, 0.453]	96.44%	0
11		9	16	25.54 *	0.220	[0.124, 0.313]	41.56%	0
12		6	40	313.18 ***	0.154	[0.083, 0.233]	68.61%	20.29%
13	−0.412	45	114	2049.75 ***	0.279	[0.235, 0.321]	80.39%	15.09%

Note: 1 = empathy and secure attachment model; 2 = empathy and anxiety attachment model; 3 = empathy and avoidance attachment model; 4 = eastern culture subgroup model; 5 = western culture subgroup model; 6 = multidimensional empathy subgroup model; 7 = cognitive empathy subgroup model; 8 = affective empathy subgroup model; 9 = IRI subgroup model; 10 = BES subgroup model; 11 = empathy task subgroup model; 12 = unpublished subgroup model; 13 = published subgroup model; k = sample number; n = effect size number; *** = *p* < 0.001; * = *p* < 0.05. To ensure the robustness of the results, moderator categories with fewer than 10 sample sizes were omitted from Egger’s test.

## Data Availability

All information of studies included in our meta-anylyses were provided in Appendix A. In correspondence and requests for data should be addressed to X.X.

## Appendix A

**Table A1:** Articles included in the meta-analysis.

Author (First)	Year	Journal/University	Article Title
Panfile, T. M.	2012	Merrill-Palmer Quarterly	Attachment Security and Child’s Empathy: The Mediating Role of Emotion Regulation
You, S.	2015	Psychology in the Schools	Bullying Among Korean Adolescents: The Role of Empathy and Attachment
Shoshani	2021	Computers in Human Behavior	Video Games and Close Relations: Attachment and Empathy as Predictors of Children’s and Adolescents’ Video Game Social Play and Socio-emotional Functioning
Burnette, J. L.	2009	Personality and Individual Differences	Insecure Attachment and Depressive Symptoms: The Mediating Role of Rumination, Empathy, and Forgiveness
McGinley	2018	The Journal of Genetic Psychology	Can Hovering Hinder Helping? Examining the Joint Effects of Helicopter Parenting and Attachment on Prosocial Behaviors and Empathy in Emerging Adults
Catrinel A. Ştefan	2016	Early Child Development and Care	The Multifaceted Role of Attachment During Preschool: Moderator of Its Indirect Effect on Empathy Through Emotion Regulation
Wei, M.	2011	Journal of Personality	Attachment, Self-Compassion, Empathy, and Subjective Well-Being Among College Students and Community Adults
Lisa et al.	2020	Social Development	Toddlers’ Preference for Prosocial versus Antisocial Agents: No Associations with Empathy or Attachment Security
Yu, G.	2012	Public Personnel Management	Improving Public Service Quality from a Developmental Perspective: Empathy, Attachment, and Gender Differences
You	2016	School Psychology International	Understanding Aggression Through Attachment and Social Emotional Competence in Korean Middle School Students
Panfile, T. M.	2013	International Journal of Behavioral Development	The Influence of Attachment Security on Preschool Children’s Empathetic Concern
Schoeps	2020	PLOS ONE	The Impact of Peer Attachment on Prosocial Behavior, Emotional Difficulties and Conduct Problems in Adolescence: The Mediating Role of Empathy
Britton, P. C.	2010	The Journal of Social Psychology	The Relations Among Varieties of Adult Attachment and the Components of Empathy
Zhao, K.	2017	Chinese Journal of Ergonomics	The Effect of attachment on College Students’ Internet Altruistic Behavior: Mediating Model of Trust and Empathy
Wang, H.	2020	Henan University	A Research on The Relationships Between Peer Attachment, Empathy and Prosocial Behavior of High School Students
Zhao, X.	2015	Chinese Journal of Clinical Psychology	Mediating Effect of Empathy between Attachment and Social Skills in College Students
Shi, Y.	2020	Tanjin Nornal University	The Relationship between Parent-Child Attachment and Altruistic Behavior in High School Students: The Mediating Roles of Peer Attachment and Empathy
Yue, H.	2020	Tanjin Nornal University	Attachment and Interpersonal distress in High School students: The Mediating role of Empathy
Huang, Y.	2017	Sichan Normal University	The Influence of Parent—child Attachment on the Acceptance of “The second child” by the Only Child——The role of empathy and self–esteem
Yang, N.	2012	Zhengzhou University	The Study of Influential Factors of Adolescence’s Empathy
Song, X.	2020	Chinese Journal of Clinical Psychology	Peer Attachment and Cyberbullying Among Junior High School Students: The Mediating Roles of Empathy and Positive Attitudes toward Cyberbullying
Yang, J	2019	Community psychology research	Childhood Emotional Neglect Predicts College Students’ Empathy Ability
Shen, J.	2020	Hunan Normal University	The Influence of Attachment on Interpersonal Sensitivity of Secondary Vocational Nursing Students: the mediating role of Empathy
Ayellet Boussi	2017	Long Island University	Numbness or Social Reconnection: The Effects of Rejection on Behavior and The Role of Empathy, Attachment, Rejection Sensitivity, and Effortful Control
Gelb	2001	Pace University	The Relationship between Empathy and Attachment in The Adolescent Population
Joireman	2001	North American Journal of Psychology	Relationships Between Dimensions of Attachment and Empathy
Profe, W. B.	2021	Journal of Social and Personal Relationships	Adolescents’ Responses to The Distress of Others: The Influence of Multiple Attachment Figures Via Empathetic Concern
Ibrahim TAS	2019	Eurasian Journal of Educational Research	The Pattern of Relationship between Attachment Styles, Gaming Addiction and Empathetic Tendency among Adolescents
Kenny, M. B.	2002	Journal of Adolescence	Instrumental and Social/Relational Correlates of Perceived Maternal And Paternal Attachment in Adolescence
Laible, D.	2004	Journal of Adolescence	Pathways to Self-Esteem in Late Adolescence: The Role of Parent and Peer Attachment, Empathy, and Social Behaviors
Laible, D.	2000	Journal of Youth and Adolescence	The Differential Relations of Parent and Peer Attachment to Adolescent Adjustment.
Li, S.	2015	Personality and Individual Differences	Indirect Aggression and Parental Attachment in Early Adolescence: Examining the Role of Perspective Taking and Empathetic Concern
Llorca-Mestre	2017	Behavior and personality	Parenting Style and Peer Attachment as Predictors of Emotional Instability in Children
Murphy, T. P.	2015	The Journal of Genetic Psychology	Attachment’s Links with Adolescents’ Social Emotions: The Roles of Negative Emotionality and Emotion Regulation
Ingrid L. van der Mark et al.	2002	Social Development	Development of Empathy in Girls During the Second Year of Life: Associations with Parenting, Attachment, and Temperament
Curcio, A. L.	2016	New Zealand Journal of Australian	Predictors of Delinquency among Adolescents and Young Adults: A New Psychosocial Control Perspective
T Hünefeldt	2013	Journal of Adolescence	The Relationship between ‘ToM’ and Attachment Related Anxiety and Avoidance in Italian Adolescents
Anne Greig	2001	British Journal of Developmental Psychology	Social Understanding, Attachment Security of Preschool Children and Maternal Mental Health
Fiorenzo Laghi	2009	Social Indicators Research	Attachment Representations and Time Perspective in Adolescence
Laible, D. J.	2004	Journal of Adolescence	Pathways to Self–Esteem in Late Adolescence: The Role of Parent and Peer Attachment, Empathy, and Social Behaviors
Pyeong Hwa	2017	Fam.environ.res	The Relationship between Attachment and Children’s Friendship Network and Friendship Quality: Focusing on the Mediating Effect of Empathy
Howard Steele	2008	Attachment and Human Development	Early Attachment Predicts Emotion Recognition at 6 and 11 Years Old
anam	2013	Fwu Journal of Social Sciences	Perceived Parental Attachment and Emotional Empathy among Adolescents.
Rosnay, M. D.	2002	Attachment and Human Development50	Individual Differences in Children’s Understanding of Emotion: The Roles of Attachment and Language
Raikes, H. A.	2006	British Journal of Developmental Psychology	Family Emotional Climate, Attachment Security and Young Children’s Emotion Knowledge in a High Risk Sample
Repacholi, R.	2004	British Journal of Developmental Psychology	Attachment and Preschool Children’s Understanding of Maternal Versus Non-Maternal Psychological States
Lenna, L.	2002	Social Development	Patterns of Attachment and Maternal Discourse Effects on Children’s Emotion Understanding From 3 to 5 Years of Age
Steele, H.	2001	Social Development	Infant-Mother Attachment at One Year Predicts Children’s Understanding of Mixed Emotions at Six Years
Waters, S. F.	2010	Journal of Psychopathology and Behavioral Assessment	Emotion Regulation and Attachment: Unpacking Two Constructs and Their Association
Lee	2018	Family and Environment Research	The Relationship between Attachment and Children’s Friendship Network and Friendship Quality: Focusing on the Mediating Effect of Empathy
